# Clinical Management of Tuberous Sclerosis: A Nephrologist's Perspective

**DOI:** 10.34067/KID.0000000904

**Published:** 2025-06-19

**Authors:** Jielu Hao Robichaud, Humayra Afrin, Vicente E. Torres, Navin R. Gupta

**Affiliations:** 1 Division of Nephrology and Hypertension, Mayo Clinic, Rochester, Minnesota; 2 Department of Biochemistry and Molecular Biology, Mayo Clinic, Rochester, Minnesota

**Keywords:** cystic kidney, genetic renal disease, genetics and development, human genetics, molecular genetics

## Abstract

Tuberous sclerosis complex (TSC) is a highly variable autosomal dominant disease characterized by dysregulated organ development and growth. Benign tumors, termed hamartomas, may occur across organ systems but typically involve the kidney, brain, skin, heart, and lung. The diagnosis, surveillance, and clinical management of TSC requires a multidisciplinary approach, adopted by dedicated multispecialty centers worldwide. Nephrology involvement predominantly stems from the morbidity and mortality related to the prototypical kidney lesion, angiomyolipomas, whose presence and degree confers risk of CKD, hypertension, retroperitoneal bleeding, and possibly renal cell carcinoma. Surveillance of kidney structural lesions, kidney function, and BP may enable early interventions that limit kidney-related morbidity and mortality, such as mammalian target of rapamycin inhibitor therapy. We review the epidemiology, genetics, and pathogenesis of TSC and how these inform the evaluation, diagnosis, and clinical management of TSC from the vantage point of the treating nephrologist.

## Introduction

Tuberous sclerosis complex (TSC) is a rare autosomal dominant disorder that afflicts 1:6000–1:10,000 live births and is caused by pathologic loss-of-function (LoF) variants in either TSC complex subunit 1 (*TSC1*) or TSC complex subunit 2 (*TSC2*).^1,2^ TSC is characterized by pleomorphic benign tumors of multiple organ systems, including the kidney, brain, skin, heart, and lung. These benign tumors, or hamartomas, are disorganized tissue comprised of cells that normally occur in the resident tissue, suggesting a developmental error.^3^ While the masses are typically noncancerous, subsequent disease manifestations may confer significant morbidity and potential mortality to necessitate early diagnosis, lifelong surveillance, and prompt clinical management.

## Epidemiology

In TSC, there is a high degree of phenotypic variability in terms of the severity and number of disease manifestations, with only 29% of patients demonstrating the Vogt diagnostic triad of facial angiofibromas, seizures, and intellectual disability.^4^ Although TSC has a high degree of penetrance, the percentage of patients with a given genotype who manifest the associated phenotype, there is also highly variable expressivity, meaning that patients with a similar genotype may have markedly different phenotypic degrees. Despite having an identical disease-causing variant, intrafamilial variability in expressivity among patients with TSC may decouple genotype and phenotype to suggest disease modification by other genes, environmental factors, or happenstance. The latter was further highlighted by a case of monozygotic twins raised in the same household differing in their severity of epilepsy and cognitive abilities.^5^ TSC in infancy and childhood most often presents with seizures.^6,7^ A retrospective study of 243 patients with TSC reflected an age at diagnosis of 0–73 years, with an average of 7.5 years and >80% diagnosed before 10 years. Conversely, presentations in adulthood are generally mild or asymptomatic, most likely due to somatic mosaicism when sporadic or due to highly variable expressivity when familial.^7^

## Genetics and Pathogenesis

**Figure 1:**
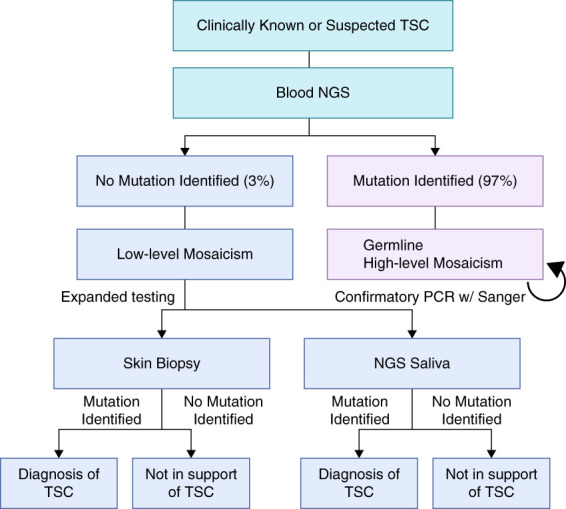
**Workflow illustrating the genetic testing strategy for the diagnosis of TSC.** For clinically diagnosed patients, NGS of blood is performed to identify pathogenic mutations. If no mutation is detected in blood, targeted genetic testing of affected tissue is recommended to improve diagnostic yield. NGS, next-generation sequencing; TSC, tuberous sclerosis complex.

TSC is caused by pathologic LoF variants in the *TSC1* and *TSC2* loci on chromosomes 9q34 and 16p13, respectively.^8,9^ *TSC1* encodes hamartin, and the gene product of *TSC2* is tuberin. Hamartin (*TSC1*), tuberin (*TSC2*), and TBC1 domain family member 7 (*TBC1D7*) form the TSC, which suppresses activation of mammalian target of rapamycin complex 1 (mTORC1).^10^ Although LoF variants in *TSC1*, *TSC2*, or *TBC1D7* each enhance mTORC1 activity, only variants in *TSC1* and *TSC2* cause TSC.^11^ LoF variants in *TBC1D7* are associated with an autosomal recessive macrocephaly/megaloencephaly syndrome, with variable phenotypes serving to highlight the complexity of mammalian target of rapamycin signaling.^12^ Mutational analysis in a cohort of 136 patients with TSC identified 75 *TSC2* variants (55.1%), 36 *TSC1* variants (26.4%), and 23 (16.9%) genetically unresolved.^13^ This study used conventional genetic testing, which detects culprit variants in 85%–90% of patients identified by diagnostic criteria, while next-generation sequencing increases detection rates to >90%.^14,15^ In prior studies, most patients with TSC who remained genetically unresolved are suspected to have intronic variants or somatic mosaicism. Next-generation whole-genome sequencing applied to blood, saliva, and/or skin tumor biopsies from 53 genetically unresolved cases identified causative *TSC1* or *TSC2* variants in 45 patients (85%). Among these 45 patients, 17 variants had an allelic frequency of <5%, five at <1%, and two only identified on skin tumor biopsies.^14^ A genetic testing workflow, with sample preparation and shipping information available at Mayo Clinic Laboratories, is outlined in Figure 1.

*TSC2* and *PKD1* are adjacent on chromosome 16p13. A *TSC2/PKD1* contiguous gene syndrome involving partial or complete deletion of both genes is associated with severe disease. An acquired somatic mutation superimposed on a germline *TSC2* mutation has been detected in kidney and pulmonary tumors, in support of a second hit hypothesis that triggers tumor formation.^16^ A similar mechanism has been reported in *PKD1* to suggest chromosomal instability at 16p13. A cohort of 325 patients with TSC indicated that 257 patients (79%) had *de novo* disease, whereas 68 patients (21%) were familial.^17^ *De novo* patients are two times more likely to bear *TSC2* variants and simplex patients 3.4 times, whereas *TSC1* and *TSC2* are equally prevalent in familial patients.^18^ Explanations for the preponderance of nonfamilial disease include (*1*) subclinical parental somatic mosaicism, (*2*) a *de novo* pathogenic variant occurring in gametes before fertilization, or (*3*) the affected patient developing somatic mosaicism during early cell divisions in embryogenesis. Each of these events would reflect the susceptibility of the TSC genomic loci to spontaneous mutation, *TSC2* (16p13) more so than *TSC1* (9q34). *TSC2* pathogenic variants are associated with more severe neurologic phenotypes, a greater risk of renal malignancy, and more frequent characteristic skin lesions. Genetic analysis of pure familial patients (*i.e*., lacking germline mosaicism) removes the phenotypic confounding of mosaicism, demonstrating that missense changes in the same amino acid may be associated with mild disease (R905Q) versus a more severe phenotype (R905W or R905G).^19^

Approximately 3600 pathogenic or likely pathogenic variants, including in-frame and out-of-frame insertions and deletions, nonsense, and missense variants have been reported in TSC with a 3:1 ratio of *TSC2*:*TSC1*. The degree of genetic variance supports the historical *de novo* nature of disease, as opposed to a predominant germline variant originating in a common generationally distant index patient. Importantly, thousands of rare *TSC1* and *TSC2* variants have been identified in a large group of adults who are unlikely to have a diagnosis of TSC, such that the determination of pathogenicity may not be straightforward and genetic testing should not be conducted in the absence of clinical suspicion.^20^

## TSC Clinical Features

### Kidney Manifestations

Typical renal manifestations of TSC include angiomyolipomas (AMLs), kidney cysts, and potentially renal cell carcinoma (RCC). A retrospective review of 167 patients with TSC found that kidney lesions occurred in 96 (57.5%), of which AML occurred in 82 patients (49.1%), cysts in 43 patients (25.8%), and RCC in four patients (2.4%).^13^ Yet, the presence of kidney lesions increases with age to affect up to 80% of patients during their lifetime.^21^ While kidney involvement is quite common, most patients remain asymptomatic and lack disease-related complications.^22^

#### AMLs

**Figure 2:**
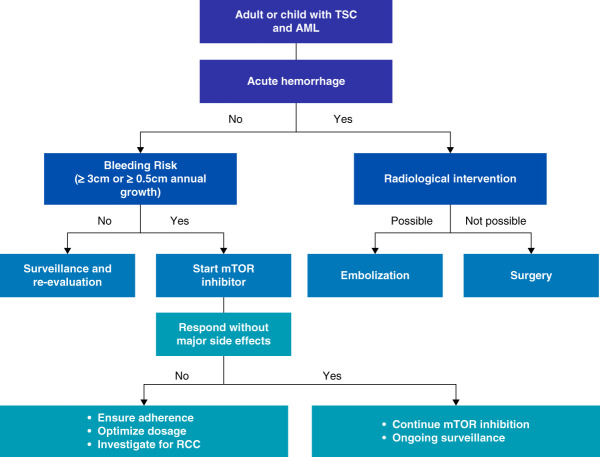
**Schematic representation of treatment strategies for AML-related bleeding.** Patients at risk of bleeding are managed with radiological interventions, while those without active bleeding risk are considered for surveillance and mTOR inhibitor therapy. AML, angiomyolipoma; mTOR, mammalian target of rapamycin; RCC, renal cell carcinoma.

AMLs, the prototypical kidney lesion, are composed of disorganized blood vessels, smooth muscle, and adipose tissue and belong to a family of masses termed perivascular epithelioid cell tumors, or PEComas.^23^ In the kidney, AMLs are believed to arise from the clonal proliferation of the pericytes that line renal blood vessels.^24^ Importantly, the prevalence of isolated kidney AMLs seems to be 20-fold greater than the prevalence of TSC, meaning that most patients with AMLs lack TSC.^23^ Among patients with TSC, AMLs are more frequent and numerous in women than men, affecting 63% of girls and 41% of boys older than 5 years, suggesting a hormonal element to their development.^25^ By pathology, immunohistochemical expression was 100% for the estrogen receptor *β*, 28% for the estrogen receptor *α*, and 38% for the progesterone receptor in AMLs.^26^ The hormonal changes and increased circulating blood volume of pregnancy have been associated with rapid AML growth and rupture, with retroperitoneal hemorrhage threatening maternal and fetal lives.^27^ AMLs may present with bleeding complications, including hematuria, versus mass effects of flank fullness/discomfort, hypertension, or obstructive nephropathy. Yet most affected patients remain asymptomatic, with most AMLs coming to attention on surveillance imaging, as retrospective cohort studies suggest that only 6.0%–9.4% of patients have symptoms related to kidney disease, most commonly hematuria.^28,29^ As AMLs ≥4 cm may develop aneurysms that rupture, guidelines advise that demonstratably enlarging AMLs of ≥3 cm be preemptively treated.^30^ Wunderlich syndrome, a rare, atraumatic, and life-threatening subcapsular hemorrhage, is most commonly caused by renal AMLs.^31^ An algorithmic approach outlines the surveillance and treatment of AML-related bleeding (Figure 2).

On the basis of composition, AMLs may be classified as classic, fat-poor, or epithelioid. Fat-containing classic variants are the most favorable, with fat content inversely proportional to symptoms or complications. Conversely, fat-poor AMLs may be indistinguishable from RCC, and their vascularity, overall size, and aneurysmal degree are associated with an increased risk of rupture.^32,33^ Fat-poor variants are present in one third of patients and often not detected by ultrasound. Guidelines recommend magnetic resonance imaging (MRI) over computed tomography to limit cumulative ionizing radiation.^34^ Rarer epithelioid variants are reported to have potential for malignant transformation, distant metastasis, and mortality.^27,35^ Yet, a histologic review of 194 TSC-related AMLs included 15 patients (7.7%) with primarily epithelioid variants (>50%), which were not associated with local recurrence or distant metastasis at a mean follow-up of 5.1 years, to reflect a benign course after resection.^35^ Meanwhile, a smaller study of 33 histologically pure epithelioid variants (100%) found local recurrence and metastasis in 17% and 49%, yet only nine were reported to have TSC.^36^ Whether TSC confers a higher risk of RCC remains debatable, with confounding from increased identification of indolent masses due to surveillance imaging and difficulties in distinguishing complex, fat-poor AMLs from RCC.^34^

#### Cysts

Simple cysts are the second most common kidney manifestation of TSC with a reported incidence of nearly 50%.^21,37^ Most patients have a limited number of small cysts that remain asymptomatic. Those with *TSC2/PKD1* contiguous gene syndrome generally present with severe cystic disease and renal insufficiency in their first year of life.^38–40^ In a study of 17 such patients, all reached ESKD by age 29 years with hypertrophic kidneys that resembled advanced autosomal dominant polycystic kidney disease (PKD), while five were incorrectly diagnosed as autosomal recessive or early-onset autosomal dominant PKD due to cysts preceding other TSC features.^40^

#### RCC

The prevalence of RCC in TSC has been reported as 1.4%–4% in multiple studies.^34^ However, this is not dissimilar from the lifetime prevalence of RCC of 1.3%–2.3% in the general population of 1.3%.^41^ Reported TSC-associated RCC has been characterized as papillary, oncocytic chromophobe, or granular eosinophilic macrocystic variants.^42,43^ The risk of RCC seems to be higher in *TSC2* mutants than *TSC1*, particularly for patients with *TSC2/PKD1* contiguous gene syndrome. Patients with TSC develop clinically recognized RCC at an earlier age than the general population,^44^ yet this finding is likely to be confounded by lead-time bias derived from frequent intra-abdominal surveillance imaging. In addition, the often complex histology of benign AMLs may be difficult to discriminate from RCC, making the burden of RCC in patients with TSC difficult to define.

#### CKD

A subset of patients with TSC develop CKD, which progresses to ESKD, although 40% of patients with TSC develop CKD (GFR <60 ml/min) 30 years before the general population.^45^ Progressive CKD has been attributed to hyperfiltration caused by the loss of nephrons from tumor invasion or surgical interventions.^46^ CKD typically manifests subnephrotic range proteinuria, with a secondary FSGS pattern of injury on biopsy. The presence of enlarging AMLs or degree of macrocystic disease has been linked to ESKD^47^; however, the reported overall incidence of ESKD may be as low as 1%.^48^ *TSC2* pathogenic variants are more associated with CKD and the female-to-male ratio of CKD in patients with TSC was 2:1, with a mean age of 29 years, in a French national survey.^46^

## Evaluation and Diagnosis

TSC is an inherited neurocutaneous disorder, with a highly variable degree of benign hamartomas in the kidneys, heart, and lungs. Clinical suspicion should be heightened in patients with prenatal cardiac rhabdomyoma(s), infantile spasms/epilepsy, and childhood hypomelanotic skin macules. However, each of these clinical features are nonspecific in isolation, and features of TSC may not present simultaneously. Rather than a diagnostic odyssey, genetic testing identifies disease-causing *TSC1* or *TSC2* variants in up to 90% of patients who eventually fulfill the diagnostic criteria.^13,14^ A molecular diagnosis is not required for individuals who meet clinical criteria for TSC. Although not required for diagnosis, genetic testing should be offered to those who fulfill TSC criteria as a result of prognostic and familial utility.

### Genetic Diagnostic Criteria

In 2013 and 2021, the International TSC Consensus Group reviewed their diagnostic criteria and surveillance and management recommendations, with notable updates pertaining to the inclusion of genetic testing and highlighting the frequency of genetic mosaicism.^2,49^ The molecular identification of a pathogenic *TSC1* or *TSC2* variant by the standards of the American College of Medical Genetics is diagnostically sufficient for TSC. Genetic testing should be offered to all patients with TSC and to all first-degree relatives of index patients because the identification of a pathogenic *TSC1* or *TSC2* variant is sufficient for diagnosis. The spatiotemporal variability of disease manifestations, in terms of both patient age and organ involvement, signify the importance of such practice for early diagnosis, surveillance, and management.^2^ Although this practice differs from a recent expert consensus statement, which does not recommend the routine genetic analysis of family members of TSC patients with a known pathogenic variant, their recommendation was level D (weak) and conceded that their approach may not be cost-effective.^34^

### Clinical Diagnostic Criteria

The clinical criteria of TSC were updated in 2021 and include 11 major criteria and seven minor criteria. A definitive clinical diagnosis is made with either two major features or a combination of one major and two minor features^2^ (Table 1).

**Table 1:** Tuberous sclerosis clinical criteria

Organ System	Major Criteria (11)	Minor Criteria (7)
Skin	Angiofibroma (≥3) or fibrous cephalic plaque	Confetti skin lesions
	Hypomelanotic macules (≥3 of ≥5 mm in diameter)	
	Ungual fibromas (≥2)	
	Shagreen patch	
Brain	Subependymal nodule (≥2)	
	Subependymal giant cell astrocytoma	
	Multiple cortical tubers and/or radial migration lines	
Kidney	AMLs (≥2)	Multiple kidney cysts
Lung	Lymphangiomyomatosis	
Heart	Cardiac rhabdomyoma	
Eyes	Multiple retinal hamartomas	Retinal achromic patch
Bone		Sclerotic bone lesions
		Dental enamel pits (≥3)
Oral		Intraoral fibromas (≥2)
Miscellaneous		Nonrenal hamartomas

AML, angiomyolipoma

## Renal Surveillance and Management

Nephrology management of TSC relates to the detection, surveillance, and treatment of renal lesions (AMLs, kidney cysts, and RCC) and their potentially related sequelae of CKD, proteinuria, and hypertension. Although intellectual disability, epilepsy, and skin manifestations may be severe and life-altering, associated kidney disease is the most common cause of death in adults with TSC.^50^ In 2024, a consensus statement was published from the European Rare Kidney Disease Reference Network regarding the clinical management of kidney involvement in TSC, using input from nephrologists, urologists, radiologists, geneticists, pathologists, and patients themselves.^34^

### Renal Lesions (AMLs, Cysts, and RCC)

Expert consensus recommends an abdominal MRI at diagnosis and annually thereafter, with reduced frequency to every 2–3 years for small lesions (<1 cm) that demonstrate chronic stability (≥3 years). As the development of kidney manifestations may be delayed, noting median ages for cyst and AML detection are 3 and 8–13 years, respectively, regular surveillance should not be abandoned even in the absence of kidney lesions.^34^ Because TSC is often diagnosed in childhood and kidney manifestations are delayed, patients require bridging between a pediatric and adult nephrologist independent of preexisting kidney lesions. MRI is the modality of choice because it adequately discerns the soft tissue components of AMLs, noting fat-poor variants may be isoechoic and difficult to visualize by ultrasound while surveillance CTs may lead to significant cumulative radiation exposure. In Examining Everolimus in a Study of Tuberous Sclerosis Complex (EXIST-2), a multicenter randomized, placebo-controlled phase 3 trial of 118 TSC adults with renal AMLs, treatment with the mTORC1 inhibitor everolimus achieved ≥50% reduction in AMLs of ≥3 cm in 42% of participants by 3 months and 55% at 6 months, as compared with 0% in the placebo arm, whereas 80% of patients overall had >30% reduction at 6 months.^51,52^ Meanwhile, in Examining Everolimus in a Study of Tuberous Sclerosis 1, everolimus induced a ≥50% reduction in subependymal giant cell astrocytoma volume in 35% of patients, compared with 0% in placebo.^53^ Limiting the size and growth of AMLs may reduce their hemorrhagic risk because the vessels of AMLs lack a complete elastic layer and appear predisposed to microaneurysms and spontaneous bleeding.^54^ Prospective studies of interventions that reduce the risk or severity of AML bleeding are poorly feasible because the incidence may be as low as 5%.^55^ Retrospectively defined risk factors of retroperitoneal hemorrhage or the need for nephrectomy or embolization include AML size ≥3 cm, increased AML vascularity, presence of microaneurysms >5 mm, exophytic AML growth, *TSC2* pathogenic variant, female sex, and elevated body mass index.^34^ Aside from bleeding, AMLs have been somewhat associated with RCC, as previously discussed. Given that fat-poor AMLs frequently occur in patients with TSC and imaging cannot definitively distinguish them from RCC, histologic sampling may be restricted to AMLs manifesting rapid and sustained growth of >0.5 cm per year, particularly those which do not respond to mTORC1 inhibition.^56^ Although the surgical management of nonhemorrhagic AMLs is often beyond the control of the treating nephrologist, a cautious approach is justified.

### CKD

As compared with the general population, adults with TSC are at increased risk of progressive CKD.^47^ Children with TSC rarely develop CKD, even in the setting of a significant burden of renal lesions, and the monitoring of kidney function can be deferred until annual measurements in adulthood.^34^ While mTORC1 inhibition generally shrinks AMLs, a secondary analysis of the 235 patients with TSC in Examining Everolimus in a Study of Tuberous Sclerosis 1 and EXIST-2 did not show that everolimus slowed CKD progression.^57^ However, pooled data from 621 patients with TSC suggested that treatment with mTORC1 inhibitors may reduce the rate of nephrectomies and associated CKD.^58^ Given that the risk of CKD is dictated by the AML burden, the expert consensus is to use mTORC1 inhibitor therapy to prevent enlarging lesions from becoming >3 cm.^34^ Meanwhile, mTORC1 inhibitor therapy can induce or exacerbate proteinuria, which may become refractory with extended treatment, such that eGFR and proteinuria should be checked before starting treatment and monitored every 3–12 months.^57^ There is no current evidence that the treatment approach to CKD and proteinuria requires alteration in TSC.

### Hypertension

Patients with TSC have a heightened risk of hypertension, which increases with age. The childhood prevalence of hypertension is estimated at 5% and adulthood prevalence at 25%.^55^ A standardized office BP measurement should be conducted annually and a 24-hour ambulatory BP monitoring conducted for adolescents and adults with an office BP of ≥120/70. Angiotensin-converting enzyme inhibitors or angiotensin receptor blockers are the preferable first-line antihypertensives, irrespective of the presence of proteinuria. Although studies of the optimal BP in TSC are lacking, patients with *TSC2/PKD1* contiguous gene syndrome should be maintained at a systolic BP target of 95–110 on the basis of the Kidney Disease Improving Global Outcomes Clinical Practice Guidelines for Autosomal Dominant PKD.^59^

### Treatment

Kidney-related indications for mTORC1 inhibition aim to reduce bleeding complications, prevent nephrectomies, and maintain kidney function. Importantly, mTORC1 inhibition is preventative therapy for spontaneous bleeding from AMLs but has no role in the acute management of life-threatening hemorrhage, such as Wunderlich syndrome, with renal artery embolization highly preferred over nephrectomy as the first approach. Everolimus has been studied in this context and has been advised for enlarging AMLs of >3 cm independent of age, whereas sirolimus may be a reasonable alternative.^34^ Typically, AMLs responded to mTORC1 inhibitors by 3 months in EXIST-2, and AML volume maximally reduced within 6–12 months.^60^ The safety and efficacy of everolimus have been demonstrated for up to 4 years of treatment.^52^

## Family Planning

In addition to a multidisciplinary approach, geneticists, genetic counselors, and reproductive specialists participate in patient care. Prospective parents should have informed consent regarding the risks of germline transmission, which may be highly variable in both likelihood and severity. Given the frequency of somatic mosaicism, gonadal involvement may be defined by genetic testing of the sperm or egg from an affected parent. If gamete genetic testing confirms a likelihood for germline transmission, then preimplantation genetic testing of embryos conceived by *in vitro* fertilization may reduce the risk. After conception, whether from *in vitro* fertilization, prenatal genetic testing may be performed by chorionic villus sampling or amniocentesis.

## Conclusion

TSC is a highly variable genetic disease and often occurs sporadically because of somatic mosaicism, which may contribute to a lack of suggestive family history in patients. While severely affected patients present in childhood with intellectual disability, epilepsy, and characteristic skin lesions, many patients present to a nephrologist with enlarging kidney lesions first recognized in adolescence or adulthood. Multifocal AMLs, particularly when associated with kidney cysts, should prompt an evaluation for additional major or minor TSC criteria to establish a diagnosis. Alternatively, genetic testing alone is diagnostically sufficient. Next-generation sequencing increases the sensitivity for low-level mosaicism to detect pathologic *TSC1*/*TSC2* variants in >90% of patients. Once a diagnosis is established, surveillance MRIs are the imaging modality of choice to discern whether AML size or growth warrants mTORC1 inhibition to reduce the risk of AML-related bleeding, which may be life-threatening should the renal capsule rupture and a contained intrarenal hemorrhage extend to the retroperitoneum. Although the association of TSC with RCC is debatable, enlarging AMLs that remain nonresponsive to mTORC1 inhibition should heighten suspicion. Additional monitoring for early-onset CKD, proteinuria, and hypertension may require early intervention, but treatment would not be in a disease-specific manner.

## Supplementary Material
