# Supplementary material for: Clinical Management of Tuberous Sclerosis: A Nephrologist's Perspective
Source: Kidney360. 2025 Jun 19;6(10):1818–25. doi: 10.34067/KID.0000000904 (PMC12778014; doi:10.34067/KID.0000000904)
Supplement: Supplementary file 1 [file kidney360-6-1818-s001.pdf]

ASN American Society of Nephrology
JASN Journal of the American Society of Nephrology
KIDNEY360 Accessing Our World From Every Angle
CJASN Clinical Journal of the American Society of Nephrology

ASN Journal Disclosure Form

As per ASN journal policy, I have disclosed any financial relationships or commitments I have held in the past 36 months as included below. I have listed my Current Employer below to indicate there is a relationship requiring disclosure. If no relationship exists, my Current Employer is not listed.

H. Afrin reports the following:
Employer: Mayo Clinic

I understand that the information above will be published within the journal article, if accepted, and that failure to comply and/or to accurately and completely report the potential financial conflicts of interest could lead to the following: 1) Prior to publication, article rejection, or 2) Post-publication, sanctions ranging from, but not limited to, issuing a correction, reporting the inaccurate information to the authors' institution, banning authors from submitting work to ASN journals for varying lengths of time, and/or retraction of the published work.

Name: Humayra Afrin
Manuscript ID: K360-2025-000457R1
Manuscript Title: Clinical Management of Tuberous Sclerosis: A Nephrologist's Perspective
Date of Completion: June 12, 2025
Disclosure Updated Date: May 28, 2025

ASN American Society of Nephrology
JASN Journal of the American Society of Nephrology
KIDNEY360 Accessing Our World From Every Angle
CJASN Clinical Journal of the American Society of Nephrology

ASN Journal Disclosure Form

As per ASN journal policy, I have disclosed any financial relationships or commitments I have held in the past 36 months as included below. I have listed my Current Employer below to indicate there is a relationship requiring disclosure. If no relationship exists, my Current Employer is not listed.

N. Gupta has nothing to disclose.

I understand that the information above will be published within the journal article, if accepted, and that failure to comply and/or to accurately and completely report the potential financial conflicts of interest could lead to the following: 1) Prior to publication, article rejection, or 2) Post-publication, sanctions ranging from, but not limited to, issuing a correction, reporting the inaccurate information to the authors' institution, banning authors from submitting work to ASN journals for varying lengths of time, and/or retraction of the published work.

Name: Navin R. Gupta
Manuscript ID: K360-2025-000457R1
Manuscript Title: Clinical Management of Tuberous Sclerosis: A Nephrologist's Perspective
Date of Completion: May 28, 2025
Disclosure Updated Date: May 28, 2025

ASN American Society of Nephrology
JASN Journal of the American Society of Nephrology
KIDNEY360 Accessing Our World From Every Angle
CJASN Clinical Journal of the American Society of Nephrology

ASN Journal Disclosure Form

As per ASN journal policy, I have disclosed any financial relationships or commitments I have held in the past 36 months as included below. I have listed my Current Employer below to indicate there is a relationship requiring disclosure. If no relationship exists, my Current Employer is not listed.

J. Robichaud reports the following:
Employer: Mayo Clinic

I understand that the information above will be published within the journal article, if accepted, and that failure to comply and/or to accurately and completely report the potential financial conflicts of interest could lead to the following: 1) Prior to publication, article rejection, or 2) Post-publication, sanctions ranging from, but not limited to, issuing a correction, reporting the inaccurate information to the authors' institution, banning authors from submitting work to ASN journals for varying lengths of time, and/or retraction of the published work.

Name: Jielu Hao Robichaud
Manuscript ID: K360-2025-000457R1
Manuscript Title: Clinical Management of Tuberous Sclerosis: A Nephrologist's Perspective
Date of Completion: May 28, 2025
Disclosure Updated Date: May 28, 2025

ASN
American Society of Nephrology
JASN
Journal of the American Society of Nephrology
KIDNEY360
Accessing Our World From Every Angle
CJASN
Clinical Journal of the American Society of Nephrology

ASN Journal Disclosure Form

As per ASN journal policy, I have disclosed any financial relationships or commitments I have held in the past 36 months as included below. I have listed my Current Employer below to indicate there is a relationship requiring disclosure. If no relationship exists, my Current Employer is not listed.

V. Torres reports the following:
Employer: Mayo Clinic; Research Funding: Mironid, Tribune Therapeutics, GSK, Regulus [all payments to Mayo Foundation for Preclinical and clinical trials and preclinical research]; Honoraria: Up to Date; Patents or Royalties: Consulting agreement with uResearch Technology and MFMER for imaging analytics for PCKD.; Repurposing of probenecid to treat PCKD.; System and method of classifying ADPKD; and Advisory or Leadership Role: International Society of Nephrology (Kaplan award committee), American Society of Nephrology (editorial board), PKD Foundation advisory board.

I understand that the information above will be published within the journal article, if accepted, and that failure to comply and/or to accurately and completely report the potential financial conflicts of interest could lead to the following: 1) Prior to publication, article rejection, or 2) Post-publication, sanctions ranging from, but not limited to, issuing a correction, reporting the inaccurate information to the authors' institution, banning authors from submitting work to ASN journals for varying lengths of time, and/or retraction of the published work.

Name: Vicente E. Torres
Manuscript ID: K360-2025-000457R1
Manuscript Title: Clinical Management of Tuberous Sclerosis: A Nephrologist's Perspective
Date of Completion: June 12, 2025
Disclosure Updated Date: June 12, 2025
