# Supplementary material for: Clinical Management of Tuberous Sclerosis: A Nephrologist's Perspective
Source: Kidney360. 2025 Jun 19;6(10):1818–25. doi: 10.34067/KID.0000000904 (PMC12778014; doi:10.34067/KID.0000000904)
Supplement: Supplementary file 2 [file kidney360-6-1818-s002.pdf]

ASN Journal Disclosure Form

As per ASN journal policy, I have disclosed any financial relationships or commitments I have held in the past 36 months as included below. I have listed my Current Employer below to indicate there is a relationship requiring disclosure. If no relationship exists, my Current Employer is not listed.

A. Chade reports the following:
Employer: University of Missouri - Columbia

I understand that the information above will be published within the journal article, if accepted, and that failure to comply and/or to accurately and completely report the potential financial conflicts of interest could lead to the following: 1) Prior to publication, article rejection, or 2) Post-publication, sanctions ranging from, but not limited to, issuing a correction, reporting the inaccurate information to the authors' institution, banning authors from submitting work to ASN journals for varying lengths of time, and/or retraction of the published work.

Name: Alejandro R. Chade
Manuscript ID: K360_2025_000545
Manuscript Title: Renal single-nuclear transcriptomics identifies novel therapeutic targets in a preclinical model of chronic kidney disease
Date of Completion: June 26, 2025
Disclosure Updated Date: June 26, 2025

ASN American Society of Nephrology JASN Journal of the American Society of Nephrology KIDNEY360 Accessing Our World From Every Angle CJASN Clinical Journal of the American Society of Nephrology

ASN Journal Disclosure Form

As per ASN journal policy, I have disclosed any financial relationships or commitments I have held in the past 36 months as included below. I have listed my Current Employer below to indicate there is a relationship requiring disclosure. If no relationship exists, my Current Employer is not listed.

A. Eirin reports the following:
Employer: Mayo Clinic, Rochester, MN

I understand that the information above will be published within the journal article, if accepted, and that failure to comply and/or to accurately and completely report the potential financial conflicts of interest could lead to the following: 1) Prior to publication, article rejection, or 2) Post-publication, sanctions ranging from, but not limited to, issuing a correction, reporting the inaccurate information to the authors' institution, banning authors from submitting work to ASN journals for varying lengths of time, and/or retraction of the published work.

Name: Alfonso Eirin
Manuscript ID: K360-2025-000545R1
Manuscript Title: Renal single-nuclear transcriptomics identifies novel therapeutic targets in a preclinical model of chronic kidney disease
Date of Completion: June 26, 2025
Disclosure Updated Date: June 26, 2025

ASN American Society of Nephrology JASN Journal of the American Society of Nephrology KIDNEY360 Accessing Our World From Every Angle CJASN Clinical Journal of the American Society of Nephrology

ASN Journal Disclosure Form

As per ASN journal policy, I have disclosed any financial relationships or commitments I have held in the past 36 months as included below. I have listed my Current Employer below to indicate there is a relationship requiring disclosure. If no relationship exists, my Current Employer is not listed.

E. Mccarthy has nothing to disclose.

I understand that the information above will be published within the journal article, if accepted, and that failure to comply and/or to accurately and completely report the potential financial conflicts of interest could lead to the following: 1) Prior to publication, article rejection, or 2) Post-publication, sanctions ranging from, but not limited to, issuing a correction, reporting the inaccurate information to the authors' institution, banning authors from submitting work to ASN journals for varying lengths of time, and/or retraction of the published work.

Name: Elizabeth Ann Mccarthy
Manuscript ID: K360-2025-000545R1
Manuscript Title: Renal single-nuclear transcriptomics identifies novel therapeutic targets in a preclinical model of chronic kidney disease
Date of Completion: June 26, 2025
Disclosure Updated Date: June 26, 2025

ASN
American Society of Nephrology
JASN
Journal of the American Society of Nephrology
KIDNEY360
Accessing Our World From Every Angle
CJASN
Clinical Journal of the American Society of Nephrology

ASN Journal Disclosure Form

As per ASN journal policy, I have disclosed any financial relationships or commitments I have held in the past 36 months as included below. I have listed my Current Employer below to indicate there is a relationship requiring disclosure. If no relationship exists, my Current Employer is not listed.

R. Sitz has nothing to disclose.

I understand that the information above will be published within the journal article, if accepted, and that failure to comply and/or to accurately and completely report the potential financial conflicts of interest could lead to the following: 1) Prior to publication, article rejection, or 2) Post-publication, sanctions ranging from, but not limited to, issuing a correction, reporting the inaccurate information to the authors' institution, banning authors from submitting work to ASN journals for varying lengths of time, and/or retraction of the published work.

Name: Rhys Sitz
Manuscript ID: K360-2025-000545R1
Manuscript Title: Renal single-nuclear transcriptomics identifies novel therapeutic targets in a preclinical model of chronic kidney disease
Date of Completion: June 27, 2025
Disclosure Updated Date: June 27, 2025

ASN
American Society of Nephrology
JASN
Journal of the American Society of Nephrology
KIDNEY360
Accessing Our World From Every Angle
CJASN
Clinical Journal of the American Society of Nephrology

ASN Journal Disclosure Form

As per ASN journal policy, I have disclosed any financial relationships or commitments I have held in the past 36 months as included below. I have listed my Current Employer below to indicate there is a relationship requiring disclosure. If no relationship exists, my Current Employer is not listed.

S. Sivasankaran reports the following:
Employer: washington University St. Louis

I understand that the information above will be published within the journal article, if accepted, and that failure to comply and/or to accurately and completely report the potential financial conflicts of interest could lead to the following: 1) Prior to publication, article rejection, or 2) Post-publication, sanctions ranging from, but not limited to, issuing a correction, reporting the inaccurate information to the authors' institution, banning authors from submitting work to ASN journals for varying lengths of time, and/or retraction of the published work.

Name: Sathesh K Sivasankaran
Manuscript ID: K360-2025-000545R1
Manuscript Title: Renal single-nuclear transcriptomics identifies novel therapeutic targets in a preclinical model of chronic kidney disease
Date of Completion: June 26, 2025
Disclosure Updated Date: June 26, 2025
